# A Domestic Secret

**Published:** 1885-09

**Authors:** 


### A Domestic Secret.

There is no large amount of human happiness outside of the married life, although there is precious little in it—in very, very rare cases. If you look at half a dozen or more of little children at play, they will every once in a while be found "spatting" with one another and apparently almost ready to scratch each other's eyes out; and in ten minutes afterwards they will be hugging each other as lovingly as two babes in the woods. And nine-tenths of the little "tilts" and "set-tos" of man and wife are as evanescent as an infant's quarrel, with a rebound of extra lovingness which more than compensates for the episode marital. But notwithstanding a calm comes after a storm, and the clear shining after a rain, it would be better if the storm matrimonial had never come, and the rain had never fallen. The reader may envy the Californian who lately wrote us after many years of married life, "My wife never gave me a cross word, never uttered a complaint, and never gave occasion for reproof." The strangest part of the thing was, his wife is still living! Widows and widowers "have a way" to "make believe" that the "departed" were semi-angelic. The severely critical will aver,--

"It is all but a dream at the best."

We will not argue the case, but for the sake of avoiding some extremely ugly things of the monitor within, now and hereafter, the JOURNAL, as is its wont, prefers to deal in preventive measures, and hereby advises as a certain means of avoiding the terrible calamity and the inevitable disgrace of "separation" or divorce, and of insuring a repetition of domestic felicities which will make the family but little short of an earthly heaven,—

KEEP YOUR MOUTH SHUT,

O man or woman, whenever the other of you is noticed to be angry in the least.

"O, my dear, the cow has swallowed the grindstone."

"I told you so," said the other. Most persons will find it less difficult to face a regiment of bayonets, than to refrain from "I told you so," when an inconsistency has been noticed or a prophecy fulfilled.

In all families there will be errors of judgment, from want of sufficient information or otherwise. Between the married pair, one is often led to do what the other would not; but as it is almost always done with the very best of motives, and with the belief that it will be for the general good, it is neither kind, or wise, or generous, or high-minded, when the error or unwisdom of it is clearly demonstrated, to follow it with a semi-malignant and cowardly crow, "I told you so."

The great secret, then, for the promotion of domestic happiness, and for the avoidance of much that is promotive of unhappiness and mental disquietude, is simply, when one of a married pair notices that the other is in an angry, or complaining or fault-finding mood, to

KEEP THE MOUTH SHUT.

That is, not the other "fellow's" but your own; the fact is, the more you let the other one do all the talking, the better for the silent one; for two processes will go on, one following the other. The first one is, the talker will get more and more enraged as the conviction appears clear of being in the right; but when the steam is all out, and the excitement over, compunctions come, and shame and self-contempt at the thought of being a fool of one's own making; and before an hour passes, the defaulter will "sidle" up to you, and will be ever so loving, and will be better and more indulgent and more liberal for a year to come. This is the way to achieve "crowning" domestic victories, more enduring than any ever achieved by the stern old Cromwell,—a victory which brings love and affection and heart kisses, and the easiest victories in the world; for all that is to be done is to *keep the mouth shut.*

